# Identification of Gender-Specific Molecular Differences in Glioblastoma (GBM) and Low-Grade Glioma (LGG) by the Analysis of Large Transcriptomic and Epigenomic Datasets

**DOI:** 10.3389/fonc.2021.699594

**Published:** 2021-09-21

**Authors:** Md Tipu Khan, Bharat Prajapati, Simran Lakhina, Mridula Sharma, Sachin Prajapati, Kunzang Chosdol, Subrata Sinha

**Affiliations:** ^1^Department of Cellular and Molecular Neuroscience, National Brain Research Centre (NBRC), Manesar, India; ^2^Department of Medical Biochemistry and Cell Biology, The Sahlgrenska Academy, Institute of Biomedicine, University of Gothenburg, Gothenburg, Sweden; ^3^Department of Biochemistry, All India Institute of Medical Science, Delhi, India

**Keywords:** glioblastoma multiforme, low-grade glioma (LGG), transcriptomics, epigenetics, weighted gene co-expression network analysis (WGCNA), pathway analysis, survival analysis, gender-specific analysis

## Abstract

Differences in the incidence and outcome of glioma between males and females are well known, being more striking for glioblastoma (GB) than low-grade glioma (LGG). The extensive and well-annotated data in publicly available databases enable us to analyze the molecular basis of these differences at a global level. Here, we have analyzed The Cancer Genome Atlas (TCGA) and Chinese Glioma Genome Atlas (CGGA) databases to identify molecular indicators for these gender-based differences by different methods. Based on the nature of data available/accessible, the transcriptomic profile was studied in TCGA by using DeSeq2 and in CGGA by T-test, after correction based. Only IDH1 wild-type tumors were studied in CGGA. Using weighted gene co-expression network analysis (WGCNA), network analysis was done, followed by the assessment of modular differential connectivity. Differentially affected signaling pathways were identified. The gender-based effects of differentially expressed genes on survival were determined. DNA methylation was studied as an indicator of gender-based epigenetic differences. The results clearly showed gender-based differences in both GB and LGG, whatever method or database was used. While there were differences in the results obtained between databases and methods used, some major signaling pathways such as Wnt signaling and pathways involved in immune processes and the adaptive immune response were common to different assessments. There was also a differential gender-based influence of several genes on survival. Also, the autosomal genes NOX, FRG1BP, and AL354714.2 and X-linked genes such as PUDP, KDM6A, DDX3X, and SYAP1 had differential DNA methylation and expression profile in male and female GB, while for LGG, these included autosomal genes such as CNIH3 and ANKRD11 and X-linked genes such as KDM6A, MAOB, and EIF2S3. Some, such as FGF13 and DDX3X, have earlier been shown to have a role in tumor behavior, though their dimorphic effects in males and females have not been identified. Our study thus identifies several crucial differences between male and female glioma, which could be validated further. It also highlights that molecular studies without consideration of gender can obscure critical elements of biology and emphasizes the importance of parallel but separate analyses of male and female glioma.

## Introduction

Sex differences in the prognosis of several cancers such as colorectal cancer (1), oral cancer (2), gastric carcinoma (3), and malignant melanoma (4) are well known. For glioma, there is a gender-related difference in incidence and survival, with the incidence being up to 1.6 times higher in males. Females also respond better to therapy (5). The difference is more pronounced for glioblastoma (GB), also known as glioblastoma multiforme (GBM), than for low-grade glioma (LGG). However, the detailed molecular differences between the sexes are still not well understood. Somewhat of an exception is the estrogen receptor family, and there are several publications on the role of these receptors in glioma (6, 7). One report also suggests that the testosterone promotes growth of glioblastoma by increasing cell invasion, migration, and proliferation in case of males, and androgen antagonists have blocked this effect in cell lines (8). A recent report has highlighted the differential response of male and female patients to chemotherapy, with female patients showing better response that was observed to be due to differences in cell cycle and integrin signaling (9). Another study, utilizing sex-specific genome-wide association study (GWAS) analysis, has reported three loci with sex-specific effects (10). They have used the GWAS data to further analyze and reported epidermal growth factor receptor (EGFR)-specific association in males and telomerase reverse transcriptase (TERT)-specific association in females in germline telomere maintenance pathway of previous reported GWAS hits (10).

However, the overall extent and nature of gender-related differences in high-grade glioma and LGG are still not clear. In this study, we have performed cross-sectional studies to identify and validate sex-specific genes and co-expression gene network modules. We examined transcriptomic and epigenetic datasets of GBM and LGG from The Cancer Genome Atlas (TCGA) and Chinese Glioma Genome Atlas (CGGA). First, we identified differentially expressed genes in males and females glioma patients. The transcriptomic data were also analyzed by the system biology tool weighted gene co-expression network analysis (WGCNA) to construct a co-expression gene network map of both males and females. We also used R tool modular differential connectivity (MDC) to identify the mean differential connectivity (MeDC) of co-expression of genes in male and female network modules. Finally, we have used Gene Ontology (GO) online platform to find out significant biological processes that are associated with the genes responsible for sexually dimorphic gene network in glioma. Analysis was done primarily on the components of TCGA dataset that are publicly available. This was verified on the CGGA dataset that however has fewer tumors and also a smaller set of genes. IDH1-based stratification has been done on the CGGA dataset, where the mutation status of key genes was available to all. The gender-associated differences in DNA methylation were also analyzed using TCGA dataset, and the biological significance of differentially expressed genes was assessed. The Kaplan–Meier survival scores of the top differentially expressed genes in males and females have been determined. Together, different transcriptomic, epigenomic, and survival approaches provided a strong group of molecular markers that specified the sex differences in glioma cancer biology.

## Materials and Methods

### Composition of The Cancer Genome Atlas and Chinese Glioma Genome Atlas Datasets and Transcriptomic Analysis

All the data were downloaded from publicly available TCGA datasets (https://tcga-data.nci.nih.gov/tcga/) and CGGA database (http://cgga.org.cn:9091/gliomasdb/). The transcriptomic profile data of male and female samples were downloaded separately from TCGA-GBM and TCGA-LGG projects (https://portal.gdc.cancer.gov/projects). TCGA portal has 56 females and 104 males from TCGA-GBM project and 288 males and 239 females from TCGA-LGG project. These data were RNA sequencing (RNA-seq) data expressed as fragment per kilobase per million (FPKM) that was produced on Illumina HiSeq 2000 sequences, which is the recommended data type for the WGCNA. Count files were also available for TCGA data. Differential gene expression analysis of male over female, for the same patients, was performed by DeSeq2 tool of usegalaxy.org platform using downloaded count files of respective TCGA GBM and LGG male and female patients. DeSeq2 output file was further annotated with human GRCh38 reference genome to find out the gene name and their chromosomal locations using Annotate DeSeq2/DEXSeq output tool of usegalaxy.org platform. Log2[fold change (FC)] and standard error of DeSeq2 result of TCGA data were used to plot the graph of the top 20 genes that were upregulated or downregulated in males over females in GBM and LGG. Here, 60,483 transcripts representing approximately 30,000 genes are available in TCGA. However IDH1 status is not available in the open platform of TCGA, and we do not have access to the restricted data.

In CGGA, the transcriptomic data are expressed as FPKM. These are 108 males and 71 females of LGG patients and 80 males and 50 females of GB patients that were downloaded. Approximately 15,000 genes are available on this database. Count files are not available on CGGA; hence, DeSeq2 analysis could not be done and only corrected T-test on FPKM values was possible. Therefore, differential expression and FC of male over female were performed using T-test with Bonferroni correction. The mutation status of key genes is available on CGGA. Therefore, these data were further stratified on the basis of IDH1 mutant and wild type to perform WGCNA to look into the effect of stratification on the persistence of sexual dimorphic network in male and female gliomas. A set of pure IDH1 wild-type tumors was also analyzed from this data set.

### Constructing Co-Expression Gene Network

To construct a co-expression gene network, we performed WGCNA (11) using normalized RNA-seq dataset downloaded from both TCGA and CGGA databases of male and female GBM and LGG samples. CGGA patients were further stratified on the basis of IDH1 status, and IDH1 wild-type tumors were studied. We constructed the gene expression networks that represent intra-gene interaction between male and female GBM and LGG.

R package for WGCNA was used to generate the co-expression networks. Before generating the networks, expression data were preprocessed to remove obvious outlier samples and samples with an excessive number of missing entries. For network generation and module detection, a matrix of Pearson’s correlations between all gene pairs was generated and then we converted this correlation matrix into adjacency matrix (unsigned) using a power function based on criterion of approximately scale-free topology. To reduce spurious connection and create a more biologically meaningful module, this adjacency matrix was transformed into a topological overlap matrix (TOM). Next, we performed clustering using TOM. For this, we used hierarchical clustering followed by Dynamic Tree Cut method (using R package *dynamicTreeCut*) to identify tightly co-regulated modules. Each module was represented by a unique arbitrary color code in the relevant figures.

### Modular Differential Connectivity

After the identification of co-expressed modules in male and female GBM and LGG, we performed MDC to quantify changes in co-expression network connectivity in modules with the same set of genes in male and female GB and LGG. In brief, MDC takes overlapping modules of genes and estimates the differential correlation among the same set of genes in two conditions. This also identifies the genes with gain of connectivity (GOC) and loss of connectivity (LOC) between two conditions subjected to statistical significance. DCGA package (12) of R was used to estimate MDC. For this, we have first generated the matrix using *design_mat*R tool, then we used *moduleDC*R tool (both *designmat* and *moduleDC* tool come under DCGA package) to estimate MDC with statistical significance for each identified module using WGCNA in male and female GBM and LGG transcriptomic data. To understand the functional significance of significant differential connectivity of modules, we assessed functional annotation using panther database (13).

### Epigenetic Analysis

DNA methylation data of males and females (n = 20 each) containing beta values were downloaded from TCGA-GBM and TCGA-LGG projects. The average beta values were calculated of both males and females in both projects. FC in beta values of genes in males over females was calculated by dividing average beta value in males of a gene with average beta values in females of that gene. These FC values were used to find out differentially methylated genes in male and female. Nonparametric Student’s t-test with two-tailed and Bonferroni correction assuming unequal variance was done to find out adjusted p-values. Differentially methylated genes with adjusted p-value (p < 0.01) were used to plot the heat map. Induced network module analysis was performed for the common differentially expressed and differentially methylated genes using ConsensusPathDB.

### Gene Ontology

The genes of the significant modules obtained after MDC were used to perform GO using GO resource (http://geneontology.org/) to find out the modules that combine to have a significant function in terms of biological component, cellular component, and molecular function.

### Kaplan–Meier Analysis

Kaplan–Meier analysis was done to find the effect of high and low expression of genes that are 1.5-fold downregulated or upregulated in males over females on the survival of patients (both males and females) using R2 database (https://hgserver1.amc.nl/cgi-bin/r2/main.cgi), a web-based genomic analysis and visualization application.

## Results

### Sex-Specific Transcriptomic Differences Are Present in The Cancer Genome Atlas Glioblastoma Multiforme and Low-Grade Glioma

**Figure 1:**
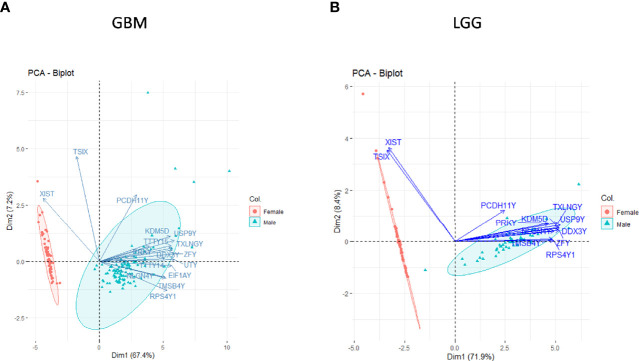
Principal component analysis (PCA) of sex-linked genes in glioblastoma multiforme (GBM) and low-grade glioma (LGG) transcriptomic data [The Cancer Genome Atlas (TCGA)]. Clear segregation of sex-linked genes in male–female **(A)** GBM and **(B)** LGG on PCA plot.

To verify whether our analysis indeed reflects the sex-specific differences in gene expression, we first assessed the segregation of genes already known to be differentially expressed by gender in TCGA dataset. The genes that are typically highly expressed (FC ≥2) in a specific sex such as the XIST, PUDP, ZFX, JPX, KDM6A, and TSIX in females and genes such as PRKY, RPS4Y2, PCDH11Y, EIF1AY, RPS4Y1, and ZFY in males were studied in GB and in LGG. We did principal component analysis (PCA) (using R package Factoextra) of the sex-specific genes of GB and LGG transcriptomic data. We found a clear segregation of data on Dim1 (PC1) and Dim2 (PC2) dimensions with respect to sex and previously known sex-specific genes (**Figures 1A, B**). This analysis provided confidence in the ability of the analysis to identify sex-specific gene expression alterations in male and female GB and LGG transcriptomic samples.

**Figure 2:**
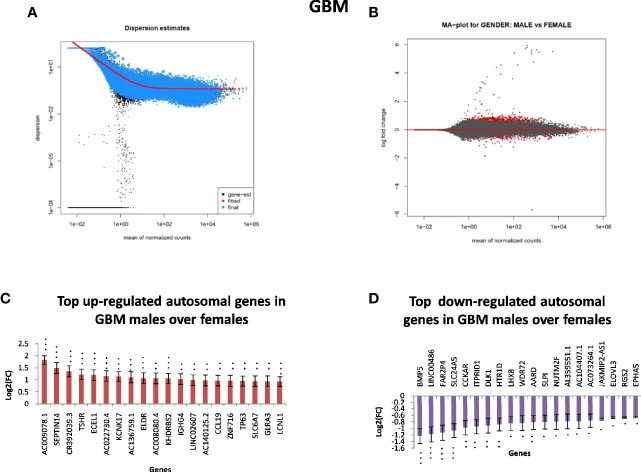
Gender-based differentially expressed genes in the transcriptomic data [The Cancer Genome Atlas (TCGA)] of glioblastoma multiforme (GBM) in males over females. **(A)** Dispersion estimate of the count values on performing DeSeq2 analysis. **(B)** MA Plot showing DeSeq2 result of the differential expression of males over females of TCGA GBM. **(C)** Graph representing top 20 upregulated genes in males over females and their Log2[fold change (FC)] values in GBM and low-grade glioma (LGG), respectively. **(D)** Graph representing top 20 downregulated genes in males over females and their Log2(FC) values in GBM 470 *p-value ≤0.05, **p-value ≤0.01, ***p-value ≤0.0001.

**Figure 3:**
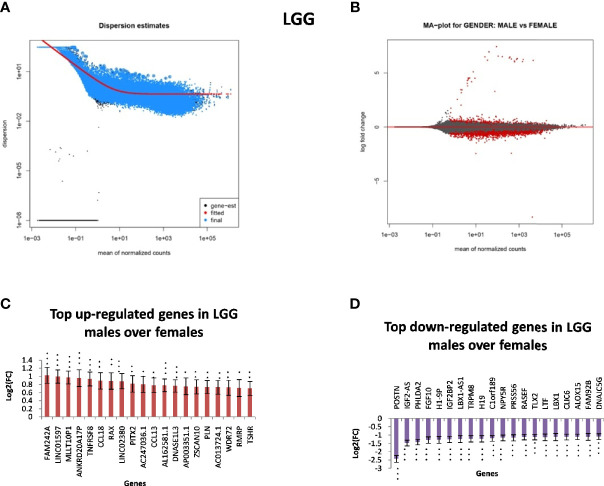
Gender-based differentially expressed genes in the transcriptomic data [The Cancer Genome Atlas (TCGA)] of low-grade glioma (LGG) in males over females. **(A)** Dispersion estimate of the count values on performing DeSeq2 analysis. **(B)** MA Plot showing DeSeq2 result of the differential expression of males over females of TCGA glioblastoma multiforme (GBM). **(C)** Graph representing top 20 upregulated genes in males over females and their Log2[fold change (FC)] values in GBM and LGG, respectively. **(D)** Graph representing top 20 downregulated genes in males over females and their Log2(FC) values in GBM 470 *p-value ≤0.05, **p-value ≤0.01, ***p-value ≤0.0001.

**Figure 4:**
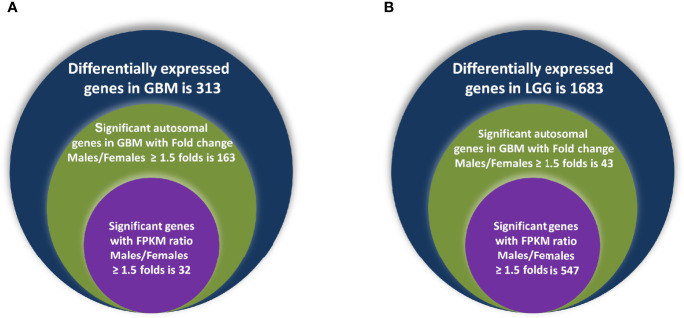
Venn diagram representing differentially expressed genes observed in glioblastoma multiforme (GBM) **(A)** and low-grade glioma (LGG) **(B)** in both genders.

In GB, further analysis showed that 313 genes were found to be significantly differentially expressed. Of these, 246 were located on autosomes and 33 genes were present on the X chromosome. We further segregated differentially expressed autosomal and X chromosome-located genes on the basis of FC. We identified that out of 246 autosomally located differentially expressed genes, 32 genes were downregulated (≥1.5-fold), and 163 genes were upregulated (≥1.5-fold) in males over females. However, out of 33 genes located on the X chromosome, 15 genes were downregulated (≥1.5-fold) and two genes (FGF13, NAP1L6P) were found to be upregulated. It is interesting that these genes are not present in the pseudo-autosomal regions (PAR1) of the X chromosome (14). In LGG, a total of 1,684 genes were found to be differentially expressed significantly (with adjusted p-value ≤0.05) in males over females. Of these, 1,564 genes were present on autosomes and 83 genes were located on the X chromosome. Out of 1,564 autosomally located differentially expressed genes, 43 genes were found to be upregulated (≥1.5-fold) and 547 genes were downregulated (≥1.5-fold) in males. However, out of those 83 genes that were present on the X chromosome, 21 genes were downregulated with FC ≥1.5-fold and two genes CD99 and AWAT2 were upregulated ≥1.5-fold. An example is the observation of the upregulation of the CD99 gene present in the Pseudoautosomal (PAR1) region in males over females. CD99 is a diagnostic marker for Ewing’s sarcoma (EWS), as it is highly expressed by these tumors (15). Top 20 genes with significant differential expression in GB and LGG are represented in **Figures 2** and **3**, respectively. The list of all the significantly differentially expressed genes in GB and LGG can be found in **Supplementary Tables S1** and **S2**, respectively. Total number of differentially expressed genes is represented with Venn diagram in **Figures 4A, B**.

The CGGA dataset (with much fewer tumors and only half the genes as TCGA) also showed significant differences in expression in both male and female tumors. However, because only FPKM values (and not counts) are available, this could not be analyzed by DeSeq2 but only by T-test with Bonferroni correction. The result of CGGA analysis showed that 25 genes were differentially expressed in GB, of which 18 were upregulated in males over females and seven were downregulated in males over female (**Supplementary Table S3**). In LGG, 26 genes were differentially expressed, with 17 being upregulated in males (**Supplementary Table S4**) of with most being common to TCGA, GBM and LGG. Most of these differentially expressed genes are on the sex chromosomes. Interestingly, CD99 is located on the PAR region of X chromosomes and is upregulated in LGG in males; in GB, the related gene closely located, CD99P1 (CD99 Antigen like 1), is upregulated. CD99 is reported as cell surface protein linked to lymphoblastic leukemia and EWS (https://www.genecards.org/cgi-bin/carddisp.pl?gene=CD99). CD99P1 has been shown to be coded by pseudo-autosomal region and has a role in cell proliferation and glioma susceptibility (https://www.genecards.org/cgi-bin/carddisp.pl?gene=CD99P1).

CGGA data were also analyzed after stratification. Only IDH1 wild-type tumors were used (as numbers of IDH1 mutated were too few). After Bonferroni correction in GB, 26 genes (**Supplementary Table S3 sheet 2**) were differentially expressed; out of these, 23 were upregulated in males and three in females. In GB, the autosomal genes upregulated in males include DCDC2B (chromosome 1), MIPEP (chromosome 13), CCDC87 (chromosome 11), HOXA1 (chromosome 7), LOC100240735 (chromosome 12), ARHGAP6 (X chromosome but upregulated in males), PDE1C (chromosome 7), ANO5 (chromosome 11), and LINC00538 (chromosome 1). Many of these genes are linked to neuromuscular development (https://www.genecards.org/). In LGG, there were 16 differentially expressed genes (**Supplementary Table S4 sheet 2**), and only 14 of these were upregulated in males. Of these, CCDC58/MIX23 (chromosome 3), ULBP2 (chromosome 6), and FAM184B (chromosome 4) were autosomal.

### Construction of Gender-Specific Transcription Modules in Glioblastoma Multiforme and Low-Grade Glioma

#### The Cancer Genome Atlas Dataset

**Figure 5:**
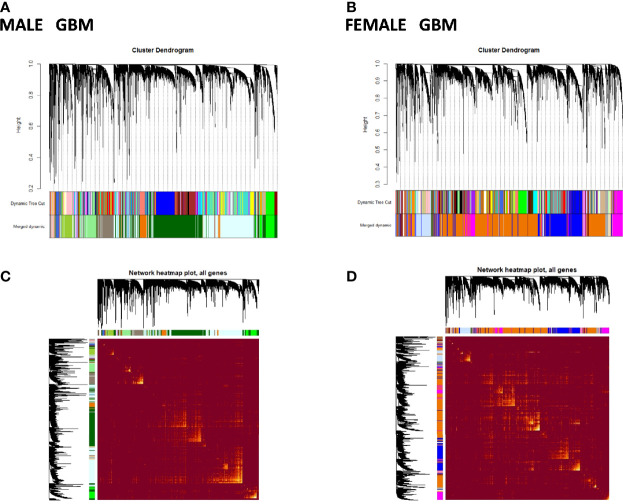
Visualization of glioblastoma multiforme (GBM) weighted gene co-expression network analysis (WGCNA): **(A, B)** Cluster dendrograms showing different modules formed in males and females in GBM. A total of 57 and 59 colors representing modules of males and females, respectively. **(C, D)** Network heat map plot of genes in different clusters in males and females in GBM. Heat map depicts the topological overlap matrix among all genes in the analysis. Dark color represents low overlap, and progressively lighter color represents higher overlap. Blocks of lighter color along the diagonal are the modules (n = 104 males, n = 56 females).

**Figure 6:**
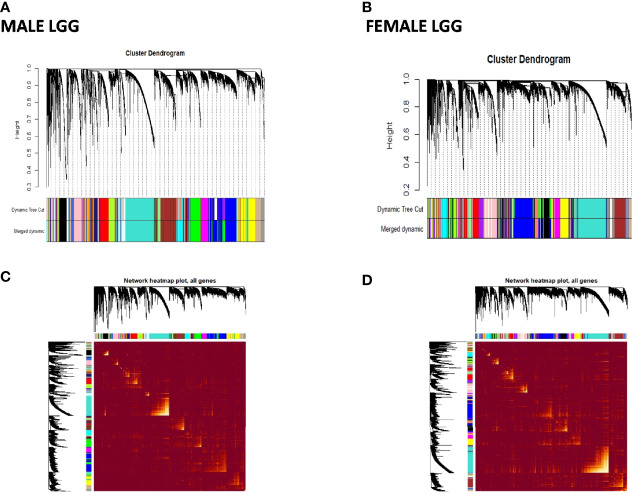
Visualization of weighted gene co-expression network analysis (WGCNA) results of low-grade glioma (LGG): **(A, B)** show cluster dendrograms of different modules formed in male and female LGG. A total of 94 and 95 colors representing modules have been identified in LGG of males and females, respectively. **(C, D)** show network heat map plot of genes of different clusters in male and female LGG, respectively. Heat map depicts the topological overlap matrix among all genes in the analysis. Dark color represents low overlap, and progressively lighter color represents higher overlap. Blocks of lighter color along the diagonal are the modules (n = 288 male and 239 female LGG).

To better understand sex-specific transcriptional changes in male and female GBM and LGG cases and to gain insight into the molecular pathways that may differ in males and females, networks of co-expressing genes were analyzed using R package WGCNA and represented as modules. Modules for both male and female GB and LGG samples were constructed for TCGA dataset. The results of clustering, dynamic branch cut, and module merging of genes in GB and LGG of male and female samples are presented in **Supplementary PDF Figures S1**–**S4**. Networks of co-expression interactome modules identified in GB and LGG of both male and female cases are represented as cluster dendogram and as network heat map plot in **Figures 5** and **6**. In GB samples of male cases, we observed 57 co-expression modules and the number of genes in each module ranged from 30 to 3,000 genes. In female GB samples, a total of 59 co-expression modules were identified, each module having 30 to 2,500 genes. Likewise, in male LGG samples, a total of 55 co-expression modules were found and the number of genes in each module ranged from 30 to 1,500 genes. In female LGG, 50 co-expression modules were observed and the number of genes in each module ranged from 30 to 3,000 genes. We could not analyze TCGA data after IDH1 stratification because of our inability to access mutation data in this set.

#### Chinese Glioma Genome Atlas Dataset

Similar to TCGA, WGCNA of CGGA patients was performed, with and without stratification based on IDH1 wild-type genotype, of the transcriptomic data of male and female GB and LGG patients into IDH1. This was done to verify gender-specific connectivity in a database other than TCGA and also to look into the effect of stratification on gender-specific connectivity. WGCNA of only IDH1 wild-type patients was performed, as the number of cases for IDH1 mutant was very low and insufficient to perform WGCNA. Wild-type IDH1 GB data consist of 48 and 25 males and females, respectively. For wild-type IDH1, LGG CGGA has data from 29 male and 19 female patients. In GB of male cases, we have observed 40 co-expression modules and the number of genes in modules ranged from 59 to 2,038 genes; in female GB, a total of 33 co-expression modules were identified, with each module having between 63 and 1,545 genes. In LGG samples of male cases, a total of 40 co-expression modules were found and the number of genes in each module ranged from 83 to 2,354 genes. In female LGG, 57 co-expression modules were observed and the number of genes in each module ranged from 40 to 3,041 genes. However, MDC after WGCNA without stratification to IDH1 wild-type does not show any significant LOC in males over females. This may be due to lesser numbers of tumors and genes in CGGA, which is insufficient to compensate for the heterogeneity. All figures can be found in **Supplementary PDF Figures S5**– **S8**.

### Gender-Specific Modular Differential Connectivity in Glioblastoma and Low-Grade Glioma

#### The Cancer Genome Atlas

To analytically detect modules with differential interconnectivity and to quantify network reorganization between males and females, we performed MDC. MDC represents the average ratio of gene network connectivity of any module in female compared to gene network connectivity of same genes in the module of male samples. This analysis identifies those modules that have either GOC or LOC between male and female modules. Statistical difference in connectivity was computed on the bases of false rate discovery (FDR), and modules with more than 5% error were excluded from further analyses.

Out of 57 modules in males and 59 modules in females found in GB samples, 22 modules showed significant LOC in GB samples of males as compared to females (**Table 1**). Module numbers 9 and 37 have been identified to have the highest LOC with MDC value of -2.27184 and -2.38596, respectively, in males over females in GB cases. In module 9, among all the genes, SAMD11, LINCO1139, and MRPL30 are identified to have maximum connectivity loss, and in module 37, the top genes showing maximum connectivity loss are HELB and CASC4 genes. Module 1 has the largest number of genes (1,155 genes) showing LOC, and module 44 has the least number of genes (27 genes) showing LOC (**Table 1**). Out of 55 modules in males and 50 modules in females found in LGG samples, 11 modules showed significant LOC in male samples as compared to female samples; four modules were approaching significance with p-value 0.06 (**Table 1**). Module numbers 46 and 43 have been identified to have the highest LOC with MeDC value of -0.15036 and -0.12776, respectively. The genes HSFY2, NIFKP3, and LOC100419861 are the top genes in module 46. Genes SEPHS1P2, RNU6-606P, and LOC100996263 are the top genes in module 43 showing maximum LOC (**Table 1**).

**Table 1:** Table showing modules having significant differential connectivity of genes in GBM and LGG.

GBM				
Module	Size	MeDC	pVal	Top LOC
mod1	1155	-1.04143	>0.0001	F2RL2, RXRG, LOC101929705
mod9	464	-2.27184	>0.0001	SAMD11, LINC01139, MRPL30
mod17	176	-0.46926	>0.0001	TSPYL2, GNRHR2, LSM11
mod21	200	-0.84427	>0.0001	FOXS1, CD34, ACVRL1
mod25	195	-1.2404	>0.0001	CCNB3, CCDC50, EPCAM
mod33	81	-0.48983	>0.0001	KCNH2, HIST1H2AE, FAM86C2P
mod36	23	-1.56159	>0.0001	CHODL, CNTNAP3B, RASGRP2
mod48	56	-0.66118	>0.0001	TSPAN9, PARP11, OGFRL1
mod52	81	-0.94843	>0.0001	TPTEP1, LOC112268292, RASSF3
mod54	35	-1.97712	>0.0001	SDCCAG8, CMAS, BLOC1S1-RDH5
mod55	60	-0.33433	>0.0001	PLD4, MYBPHL, GTSF1
mod7	308	-1.0111	0.02	LRRC66, CHIC2, DCUN1D4
mod13	1092	-1.67334	0.02	C1QTNF12, COL26A1, SLC16A8
mod20	131	-0.62816	0.02	PCDHB6, ZNF826P, EPCAM-DT
mod28	132	-0.93795	0.02	SOX3, ADRA2B, SV2B
mod29	85	-1.19756	0.02	ERO1B, MRPS16, DNAJC1
mod32	124	-0.86194	0.02	ACY3, C1orf115, HLA-A
mod37	44	-2.38596	0.02	HELB, CASC4
mod40	64	-0.7144	0.02	LGALS2, IPO9-AS1, MYBPH
mod35	109	-0.42696	0.04	LINC01206, PTPRG-AS1, FAM13B
mod39	29	-1.61494	0.04	EYS, LIMS2, BCAS1
mod44	27	-0.42343	0.04	CPM
**LGG**				
**Module**	**Size**	**MeDC**	**pVal**	**Top LOC**
mod18	351	-0.11011	0	USP9Y, CTRB1, MIR2052HG
mod34	125	-0.11337	0	RPS15AP29, LINC01098, DNAJB6P8
mod46	70	-0.15036	0	HSFY2, NIFKP3, LOC100419861
mod13	634	-0.10115	0.02	LOC339260, DNM1P46, LOC101929577
mod16	493	-0.09839	0.02	DDX3Y, RPL10P1, PNPLA1
mod22	287	-0.08038	0.02	MAJIN, LINC01606, PPIAP51
mod42	81	-0.1127	0.02	CAB39P1, ANKRD44-AS1
mod43	79	-0.12776	0.02	SEPHS1P2, RNU6-606P, LOC100996263
mod11	770	-0.0992	0.04	CT66, LINC01480, LRRC69
mod12	726	-0.10386	0.04	PAX8-AS1, PAX8, KRTAP5-10
mod19	344	-0.09355	0.04	LINC01305, POU2F3, RNU4-62P
mod14	560	-0.10249	0.06	RPL31P49,KRT8P46, TULP2
mod21	311	-0.06843	0.06	GCM1, LOC105372316, FLJ40194
mod25	214	-0.10412	0.06	BCORP1, LOC101928401, LOC339966
mod53	38	-0.13482	0.06	RNF113B

GBM, glioblastoma multiforme; LGG, low-grade glioma; LOC, loss of connectivity; MeDC, mean differential connectivity.

#### Chinese Glioma Genome Atlas

Out of 40 modules in males and 33 modules in females found in GB with wild-type IDH1, 13 modules showed significant LOC and two modules approached significance in males as compared to females (**Supplementary PDF Figure S9**). Module numbers 29 and 18 have been identified to have the highest LOC with MDC value of -0.1573 and -0.1333, respectively, in males over females in GB cases. In module 29, among all the genes, VSNL1 was identified to have maximum connectivity loss, and in module 18, the top genes showing maximum connectivity loss are STOX2, SUPV3L1, and STARD4-AS1. Module 1 has the largest number of genes (2,038 genes) showing LOC, and module 30 has the least number of genes (109 genes) showing LOC. Out of 40 modules in males and 57 modules in females found in LGG, we could not find any significant modules with LOC between males and females in CGGA IDH1 wild-type datasets (**Supplementary PDF Figures S9, S10**).

### Identification of Signaling Pathways in Glioblastoma and Low-Grade Glioma Modules With Loss of Connectivity

#### The Cancer Genome Atlas

**Figure 7:**
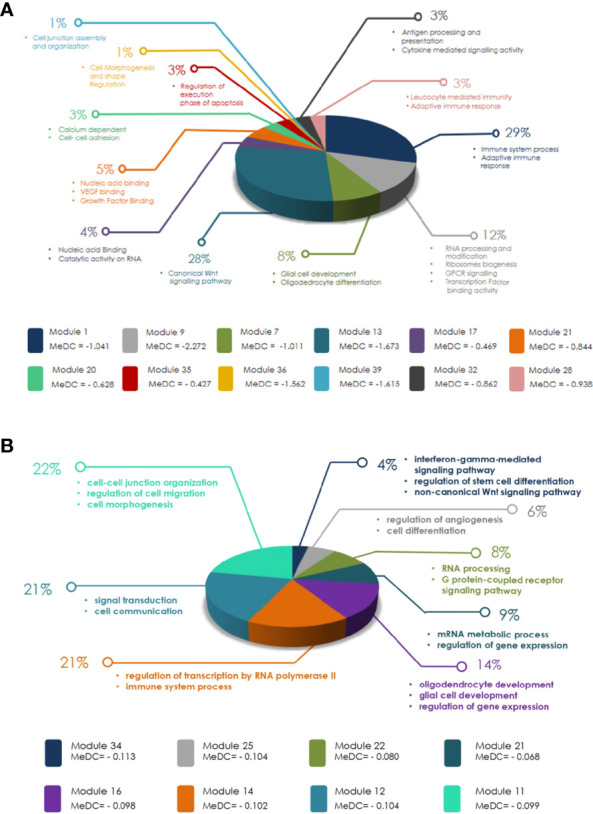
**(A)** Diagram representing modules obtained in glioblastoma multiforme (GBM) after weighted gene co-expression network analysis (WGCNA) and modular differential connectivity (MDC) analysis with significant mean differential connectivity (MeDC) values and their ontological functions. Out of 22 modules with significant MeDC values representing loss of connectivity (LOC) in males over females, only genes of 12 modules have significant ontological function as assessed by Gene Ontology (GO) database. **(B)** Diagram representing modules obtained in low-grade glioma (LGG) after WGCNA and MDC analysis with significant MeDC values and their ontological functions. Out of 15 modules with significant MeDC values representing loss of connectivity (LOC) in males over females, only genes of eight modules have significant ontological function as assessed by GO database.

To identify signaling pathways in modules having significant LOC in males over females obtained in MDC, GO analysis was done. Out of 22 male GB modules having LOC, the genes of only 12 modules formed significant signaling networks (**Figure 7A**). Among many signaling networks identified in GBM module 1, pathways playing important roles in immune system process and adaptive immune response have been identified. In addition, the canonical Wnt signaling pathway has been identified in module 13. Wnt signaling has never been looked at in a gender perspective in tumors and has not been reported in literature to date. Signaling pathways related to RNA processing and modification, ribosome biogenesis, transcription factor binding activity, and G protein-coupled receptor (GPCR) signaling have been observed in module 9.

Similarly, out of 15 (three approaching significance 0.06) LGG modules having significant LOC, only eight (three approaching significance) modules were found to have significant signaling networks (**Figure 7B**). Modules 11 and 12 are the largest modules. Module 11 ontology has shown its main function in cell–cell junction organization, regulation of cell migration, and cell morphogenesis. Module 12 showed signal transduction and cell communication. Module 16 has also shown LOC, which plays an important role in glial cell development and regulation of gene expression (**Figure 7B**). GO has also shown many other significant biological and cellular functions of these modules. The complete lists of signaling pathways identified in both GB and LGG modules are shown in **Supplementary Tables S5** and **S6**, respectively. Overall, the signaling network formed in modules of both GBM and LGG was independent of the number of genes present in the modules (**Table 2** and **Figure 7**). For example, GBM module 7 with 308 genes did not show any signaling network, while module 39 with only 29 genes formed a signaling network of biological significance on GO analysis.

**Table 2:** Table showing differentially expressed genes, their significant modules (network) obtained, and their ontological significance in males over females in GBM and LGG.

LGG GBM		
GBM significant genes upregulated in males	Significant Module No	Module ontological significance
SLC51B	mod 1	YES
OTOA	mod 13	YES
MFAP2	mod13	YES
LINC00836	mod20	YES
CD36	mod35	YES
SLC14A1	mod1	YES
GPD1	mod25	NO
YBX2	mod9	YES
TPTEP1	mod52	NO
DHRS9	mod55	NO
TP63	mod40	NO
**GBM significant genes downregulated in males**	**Significant Module No**	**Module ontological significance**
DLK1	mod13	YES
DBX2	mod32	YES
PUDP	mod13	YES
ZFX	mod13	YES
**LGG**		
**LGG significant genes upregulated in males**	**Significant Module No**	**Module ontological significance**
TG	mod46	NO
OLFM4	mod14	YES
**LGG significant genes downregulated in males**	**Significant Module No**	**Module ontological significance**
POSTN	mod11	YES
FGF10	mod21	YES
RASEF	mod11	YES
MCOLN3	mod11	YES
CHI3L1	mod11	YES
PAX2	mod34	YES
CD70	mod11	YES
EYA4	mod11	YES
CDH19	mod16	YES
HS3ST3B1	mod11	YES
ULBP3	mod11	YES
CLEC5A	mod11	YES
TMEM114	mod25	YES
P2RY2	mod46	NO
COL9A3	mod11	YES
DPEP1	mod25	YES
TARID	mod11	YES
LNCTAM34A	mod11	YES
LZTS1	mod11	YES
CXCL10	mod34	NO
MIR3681HG	mod11	YES
FBXO39	mod11	YES
THEGL	mod11	YES
CGA	mod46	NO
SH2D4A	mod11	YES
C2orf91	mod11	YES
CCN4	mod11	YES
TXLNB	mod11	YES
LURAP1L-AS1	mod16	YES
FBLN7	mod11	YES
VSTM1	mod11	YES
HABP2	mod11	YES
TSTD1	mod11	YES
LINC02432	mod11	YES
**LGG significant genes downregulated in males**	**Significant Module No**	**Module ontological significance**
MMRN1	mod11	YES
CHST8	mod11	YES
NXPH4	mod25	YES
FOXI3	mod14	YES
ETV7	mod11	YES
METTL7B	mod11	YES
CCDC8	mod11	YES
NCMAP	mod11	YES
KIAA2012-AS1	mod11	YES
MIR34AHG	mod11	YES
ARSJ	mod11	YES
TMEM61	mod11	YES

GBM, glioblastoma multiforme; LGG, low-grade glioma.

#### Chinese Glioma Genome Atlas

Chinese Glioma Genome Atlas out of 15 male GB modules in wild-type IDH1 having LOC, the genes of only 12 modules were forming significant signaling networks (**Supplementary pdf1 Figure S9**). Among the many signaling networks identified in GB module 1 are pathways playing an important role in regulation of telomere maintenance, regulation of autophagy, and interleukin (IL)6 signaling pathway (**Supplementary pdf1 Figure S10**). In addition, the canonical Wnt signaling pathway has been identified in module 11 similar to TCGA GBM result module 13.

Many of the identified ontological functions such as immune system process, Wnt signaling pathway, and cellular differentiation are common to both TCGA and CGGA databases. The complete list of signaling pathways identified in GB modules is shown in **Supplementary Table S7**.

### Gender-Specific Differences Observed in the Methylation Status of Genes

**Figure 8:**
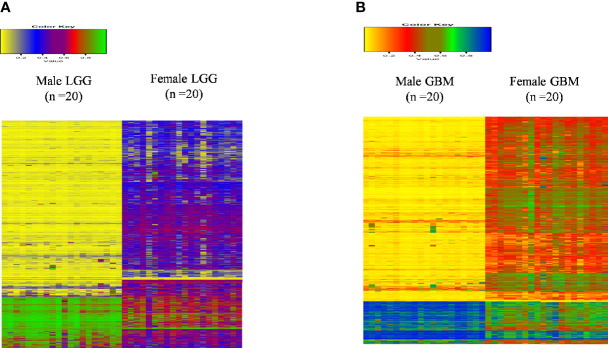
Heat map plot of the beta value of the differentially methylated genes (p-value ≤0.01) in males over females in **(A)** low-grade glioma (LGG) and **(B)** glioblastoma multiforme (GBM).

**Figure 9:**
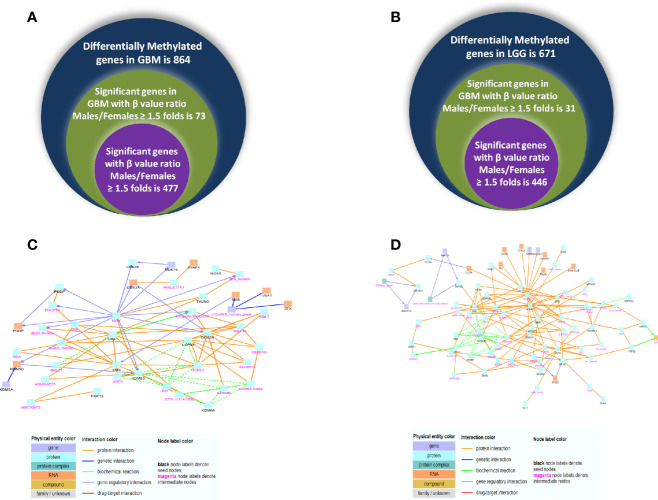
Venn diagram representing differentially methylated genes observed in glioblastoma multiforme (GBM) **(A)** and low-grade glioma (LGG) **(B)** in both genders. **(C, D)** Induced network module formed in genes differentially methylated and expressed using ConcensuspathDb, induced network module analysis tool in GBM and LGG, respectively.

Next, we analyzed the DNA methylome status of both GB and LGG samples to check for any differences in the methylation status of the genes in 20 each of male and female GBM samples as well as 20 each of male and female LGG samples. These data consist of the methylation values (beta values) of around 29,000 genes. This cohort of patients differs from the one utilized for the RNA-seq analysis, as methylation data are not available in the previous cohort and *vice versa*. On analyzing the GB samples, we have observed 864 genes to be differentially methylated, out of which 73 genes are hypermethylated (1.5-fold) and 477 genes are hypomethylated in males over females. In LGG samples, 671 genes were found to be differentially methylated. Out of these, 31 genes are hypermethylated (≥1.5-fold) and 446 are hypomethylated (≥1.5-fold) in males as compared to females. This differential methylation is represented as heat map in **Figures 8A, B**. The total number of differentially methylated genes is represented by Venn diagrams in **Figures 9A, B**.

**Figure 10:**
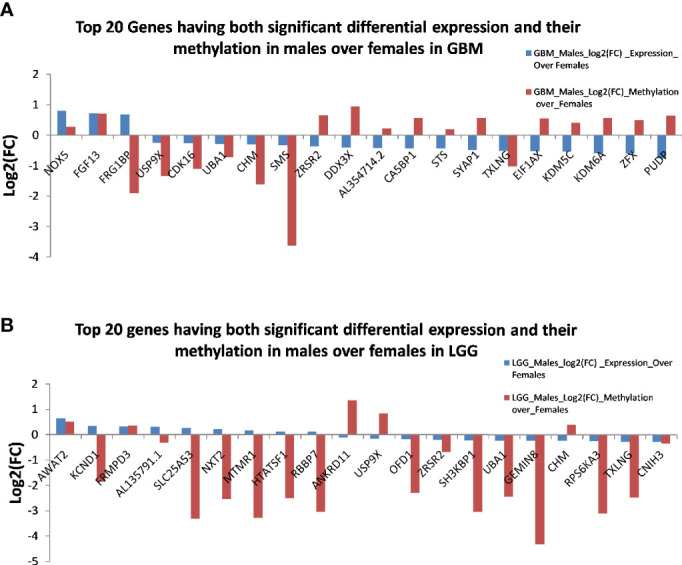
The common genes that are differentially expressed and differentially methylated in **(A)** glioblastoma multiforme (GBM) and **(B)** low-grade glioma (LGG).

Next, we correlated the differential methylation status and the differential expression level of genes in the samples of male and female GB and LGG (expression data were from another cohort). The common genes that are differentially expressed and differentially methylated genes with fold change ≥1.5 in GB (**Figure 10A**) and the common genes which are differentially expressed and methylated genes with fold change ≥1.5 in LGG (**Figure 10B**). In GB, out of 477 genes that were hypomethylated in males, only one gene was found to have upregulated expression, and out of 73 hypermethylated genes, 11 genes were found to have decreased expression. Two hypermethylated genes showed high expression and five hypomethylated genes showed low expression in **Figure 10A**. In LGG samples, out of 446 hypomethylated genes, only one gene was found to have upregulated expression, and out of 31 hypermethylated genes, only one gene was found to have decreased expression. One hypomethylated gene showed low expression (**Figure 10B**). It is possible that the results have been influenced by the two different datasets used for transcriptomic profiling and methylation. The epigenetic determinants of transcription are more complex than DNA methylation alone. Also, in published reports, there is never an absolute concordance between methylation and gene expression.

### Network Analysis Using ConcensusPathDB

Using ConcensusPathDB, we performed induced network module analysis of genes that are both differentially expressed and methylated in GB and LGG (complete list in **Supplementary Tables S8** and **S9**, respectively) in males over females. Different physical entities with types of interaction are color coded, details of which are given in **Supplementary Tables S10** and **S11**. We can speculate that this network may govern metastatic potential due to differential expression and methylation in males over female in GBM and LGG. In GB, this network sheds light on various genes that are showing maximum interactions such as DDX3X, UBA1, SMS, USP9X, and KDM5C (**Figure 9C**). In LGG, this network uncovers the genes DDX3X, RPS6KA3, SH3KBP1, and UBA1 that may act as hub genes, as they show maximum interactions with different physical entities (**Figure 9D**). Interestingly, both networks highlighted the various interactions of common gene DDX3X and UBA1 gene in both GB and LGG, showing differential methylation and expression in males over females, that are reported in a variety of cancers showing the dual roles of DDX3X and oncogenic role of UBA1 (16, 17) that can be a potential substance for study in the context of expression and methylation and its interacting partners in GBM.

### Sex-Specific Differential Influence of Genes on Survival

**Figure 11:**
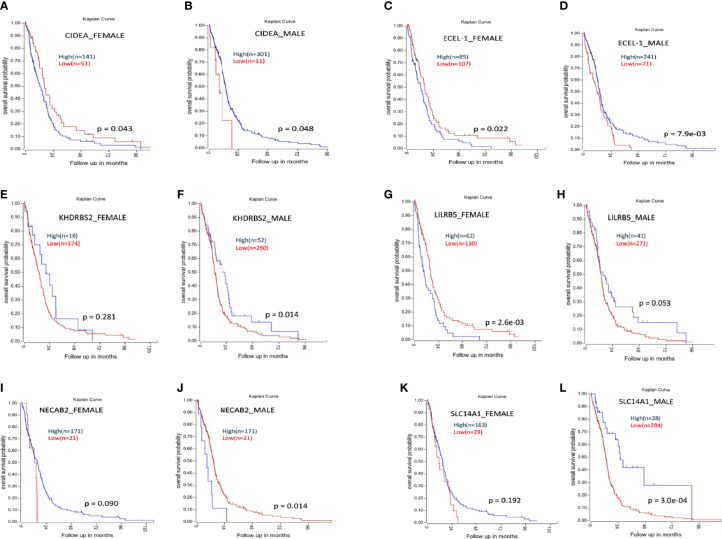
Kaplan-Meier survival analysis of some genes whose expression shows differential prognosis in males and females. **(A, B)** Higher expression of CIDEA is associated with poor prognosis in females (A) and better prognosis in males **(B)**. **(C, D)** Higher expression of ECEL-1 is associated with poor prognosis in females (C) and better prognosis in males **(D)**. **(E, F)** Higher expression of KHDRBS2 does not have any significant effect on females (E) but higher expression is associated with better prognosis in males **(F)**. **(G, H)** Higher expression of LILRB5 is associated with poor prognosis in females **(G)** and better prognosis in males **(H)**. **(I, J)** Higher expression NECAB2 do not have any significant effect on females (I) but higher expression is associated with poor prognosis in males **(J)**. **(K, L)** Higher expression of SLC14A1 does not have any significant effect on females (K) but higher expression of SLC14A1 is associated with better prognosis in males **(L)**.

Kaplan–Meier survival analysis of the top differentially expressed genes in TCGA GBM was plotted. High expression of the genes CIDEA, ECEL-1, and LILRB5 was associated with better prognosis in males, while lower expression indicated better prognosis in females. Higher expression of gene SLC14A1 showed better prognosis in males, but higher expression has no significant effect on females. Similarly, low expression of gene NECAB2 is associated with better prognosis in males, but its higher expression (with approaching significance) provided better prognosis in females (**Figure 11**).

## Discussion

Sex differences in the incidence rate and survival time have been seen in several cancers (18). Data across the globe have shown that males are at increased risk and have poor prognosis in most of the cancers (19, 20). Previously, it has been thought that the observed sex differences are due to differential exposure of males and females to environmental carcinogens (21, 22), but recent studies have shown intrinsic factors responsible for the observed sex differences in prognosis in different cancers (23–25). Many of the studies reported have analyzed the gender-based prevalence and incidence rates of cancers. However, the detailed molecular or genetic analyses are generally lacking. Gliomas are known to have higher incidence and poorer outcomes in males (26). However, the molecular basis for this is less well understood.

The estrogen-related pathway has been well studied in glioma, though it has not been possible to clinically utilize this information in the standard treatment regimens (6, 7, 27). However, there are other pathways not directly linked to sexual differentiation, which also contribute to this sexually dimorphic pattern. Expression of a few genes studied to date has shown stratified expression in males and females in GWAS (28, 29). Inhibition of adenylate cyclase activity has promoted the growth of female astrocytes but does not affect male astrocytes in a murine model of neurofibromatosis type 1 (NF1)-associated glioma (30). Association between adenylate cyclase single-nucleotide polymorphisms (SNPs) and glioma risk has been shown to be sex dependent (Warrington et al., 2015). Intracellular cAMP levels were consistently lower in males compared to female NF1-/- astrocytes. Regulation of retinoblastoma is sexually dimorphic in murine glioblastoma model upon combined loss of p53 and neurofibrin function. Loss of p53 and neurofibrin function has shown to be transforming for male but not female astrocytes (31). The current standard treatment for gliomas is more effective in females than males. Expression of cell cycle regulators is correlated with male survival, while expression of integrin signaling component is correlated with female survival (9). Therefore, devising treatment strategies with sexual differences in consideration may be expected to be more effective. In this work, we have studied whether publicly available databases could provide further evidence for differing molecular aberrations in gliomas of males and females. We have used TCGA and CGGA databases for our study. Initially, we identified the genes that are differentially expressed in males and females. Next, we used this transcriptomic data to perform WGCNA to identify clusters of highly correlated genes in males and females. We have observed the formation of significantly different clusters of genes in males and females and identified the genes responsible for these differences and the pathways in which these genes play important roles using different bioinformatic tools. As TCGA, similar studies were performed on CGGA data. WGCNA results in both databases highlight LOC in Wnt signaling and also in other processes, such as modules related to immune system processes in male GB cases. CGGA transcriptomic data consist of approximately 15,000 genes as compared to TCGA data that have transcriptomic data of around 30,000 genes. Furthermore, we have found in our network analyses that some of the pivotal pathways in cancer have gender-specific connectivity in males and females. WGCNA followed by MDC has identified many modules (i.e., a cluster of genes functioning together) that showed LOC in males as compared to females. A total of 22 such modules showed significant differences between males and females in GB. Fifteen such modules were also differentially formed in LGG. GO has shown that of the 22 modules that have differential connectivities in male over female GB, genes forming the components of 12 individual modules retain a significant ontological function. Similarly, we have found significant ontological function in five out of the 15 LGG modules. Module number 13, which shows the most prominent (in terms of the MeDC value) gender-related difference in connectivity in GB, has a role in Wnt signaling. Wnt signaling plays a very important role in development, and aberrations have been shown in a large variety of tumors, including glioma (32). To date, Wnt signaling has not been extensively studied in a gender-specific manner. Studying Wnt signaling in a gender-specific manner *in vitro* may elucidate its differential function in GB in males and females. After further WGCNA of CGGA transcriptomic data even after stratification, only IDH1 wild type showed differential connectivity in the Wnt signaling pathway.

The largest module (in numbers of component genes) with differential connectivity is module 1, which plays a role in the immune system process and adaptive immune response. Thus, this network-based study further provides an insight into targeting the glioma in gender-based manner, as both males and females have differential connectivity in various biological relevant pathways.

Our analysis using different methods has shown for the first time that autosomal genes NOX5, FRG1BP, and AL354714.2 and X-linked genes such as PUDP, ZFX, KDM6A, SYAP1, and DDX3X have been reported in different cancers to have differential DNA methylation and differential expression in males over females in GB. We have also included those genes that are expressed on sex chromosomes but are related to brain functions. However, we have not included those that are differentially expressed and are related to male or female sexually dimorphic organs and pathways. In some of the genes that we identified, a cancer-related function was already known, e.g., DDX3X has been shown to have poor prognosis in glioma (33). Similarly, FGF13, which is highly expressed in cancers such as glioma, prostate cancer, and breast cancer (34), was found to be upregulated in male GB as compared to the female counterparts. In LGG, genes such as CDKL5, KCND1, and DDX3X that have a role in different cancers have differential expression and methylation. KCND1 has been reported to have oncogenic effect in gastric cancer (35). These genes could further be validated in experimental studies to further confirm their dimorphic effect in males and females. Furthermore, Kaplan–Meier analysis in GB has also shown that some of the top differentially expressed genes also have differing effects on survival in males and females.

To conclude, our study demonstrates that differences between glioma in males and females are present, even when the analysis is done in different databases and by different methods. The genes and clusters identified under different conditions differ. However, a few pathways, e.g., those related to Wnt signaling and immune-related processes, are consistent across the databases. The study indicates that gender-based differences in glioma are ubiquitous and need to be further studied. Increased molecular stratification and experimental studies could result in more precise identification of such differences.

Molecular studies that take gender into account can thus help unravel critical elements of biology and possibly give rise to gender-specific markers for molecular classification and prognostication and for targeted therapy.

## Data Availability Statement

The original contributions presented in the study are included in the article/**Supplementary Material**. Further inquiries can be directed to the corresponding author.

## Author Contributions

MK, BP, and SS contributed to the concept and design. MK, BP, SL, MS, and SP contributed to data acquisition, analysis, and statistics. MK, BP, SL, MS, KC, and SS contributed to drafting of the manuscript. All authors contributed to the article and approved the submitted version.

## Funding

SS acknowledges JC Bose grant JCB-067/2016 from the Department of Science and Technology (DST). KC acknowledges DBT grant BT/PR13357/MED/30/1532/2015. MK, SL, and MS receive research fellowship from NBRC, CSIR, and UGC, respectively.

## Conflict of Interest

The authors declare that the research was conducted in the absence of any commercial or financial relationships that could be construed as a potential conflict of interest.

## Publisher’s Note

All claims expressed in this article are solely those of the authors and do not necessarily represent those of their affiliated organizations, or those of the publisher, the editors and the reviewers. Any product that may be evaluated in this article, or claim that may be made by its manufacturer, is not guaranteed or endorsed by the publisher.
