# Supplementary material for: Identification of Gender-Specific Molecular Differences in Glioblastoma (GBM) and Low-Grade Glioma (LGG) by the Analysis of Large Transcriptomic and Epigenomic Datasets
Source: Front Oncol. 2021 Sep 21;11:699594. doi: 10.3389/fonc.2021.699594 (PMC8491982; doi:10.3389/fonc.2021.699594)
Supplement: Supplementary file 1 [file DataSheet_1.pdf]

TCGA

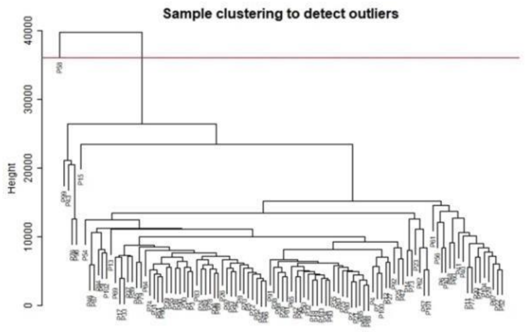

(A)

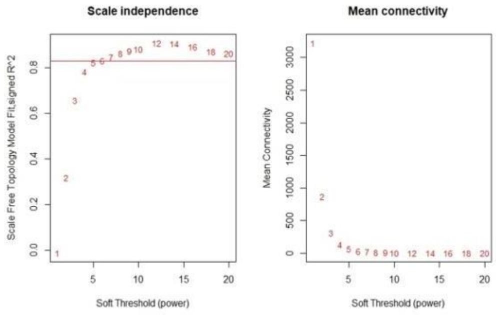

(B)

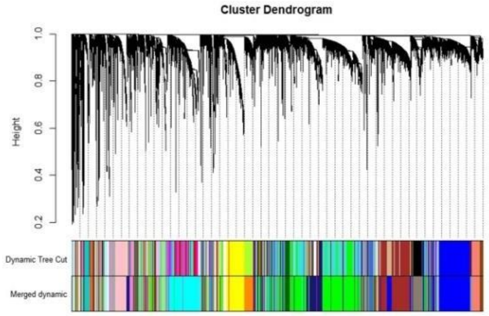

(C)

Figure S1: (A – C)

Figure S1. WGCNA network and module detection in TCGA GBM Males: A) Clustering of sample data to detectoutliers. B) Determining soft threshold and mean connectivity (β=6). C) Hierarchical clustering dendrogram of the genes based on topological overlap dissimilarity. Color coded modules ofinterconnectedgenes representsbranchesof clusteringtree.

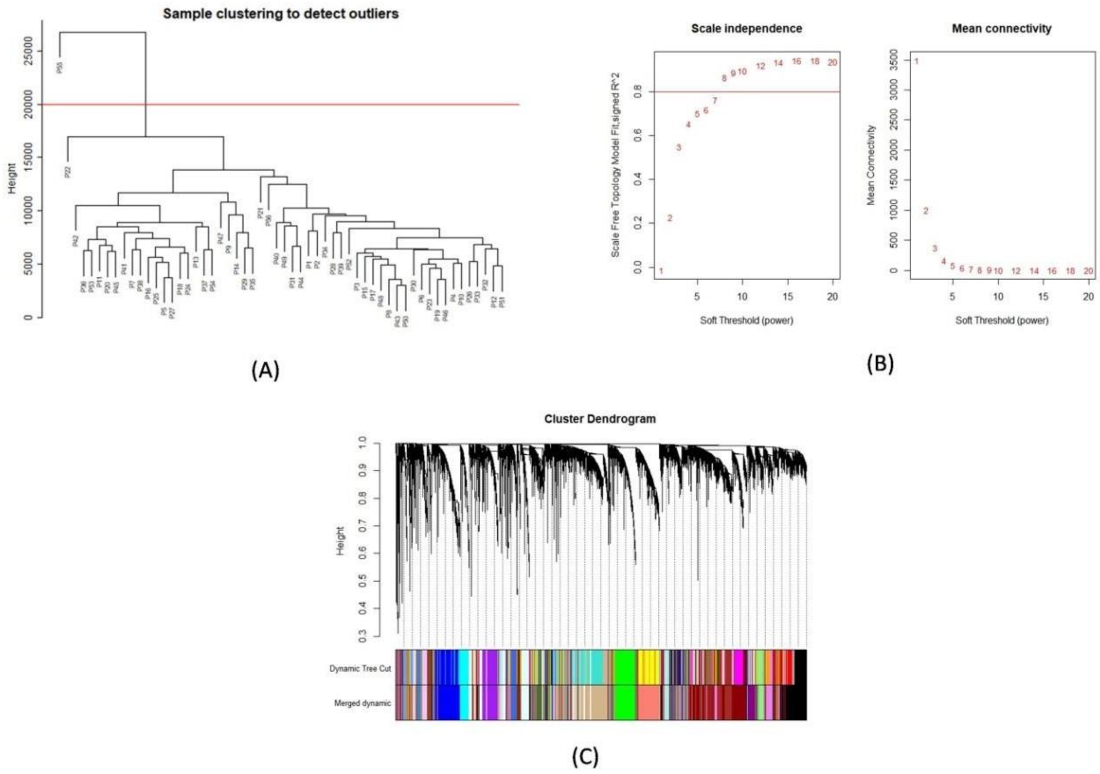

Figure S2: (A – C)

Figure S2. WGCNA network and module detection in TCGA GBM Females: A) clustering of sample data todetect outliers. B) Determining soft threshold and mean connectivity (β=8). C) Hierarchical clustering dendrogram of the genes based on topological overlap dissimilarity. Color coded modules of interconnected genes represents branches of clustering tree.

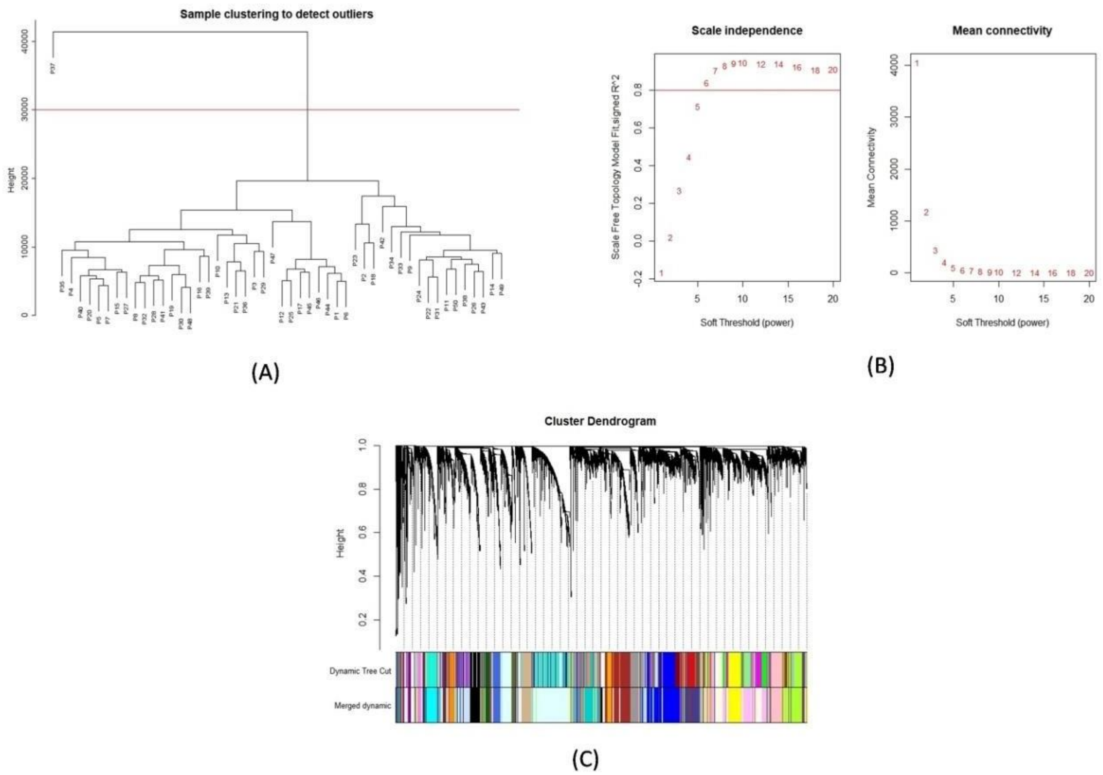

Figure S3: (A – C)

FigureS3. WGCNA network and module detectionin TCGA LGG Males:A) Clusteringofsampledatato detectoutliers. B) Determining soft threshold and mean connectivity (β=6). C) Hierarchical clusteringdendrogram of the genes based on topological overlap dissimilarity. Colour coded modules ofinterconnectedgenes representsbranchesof clusteringtree.

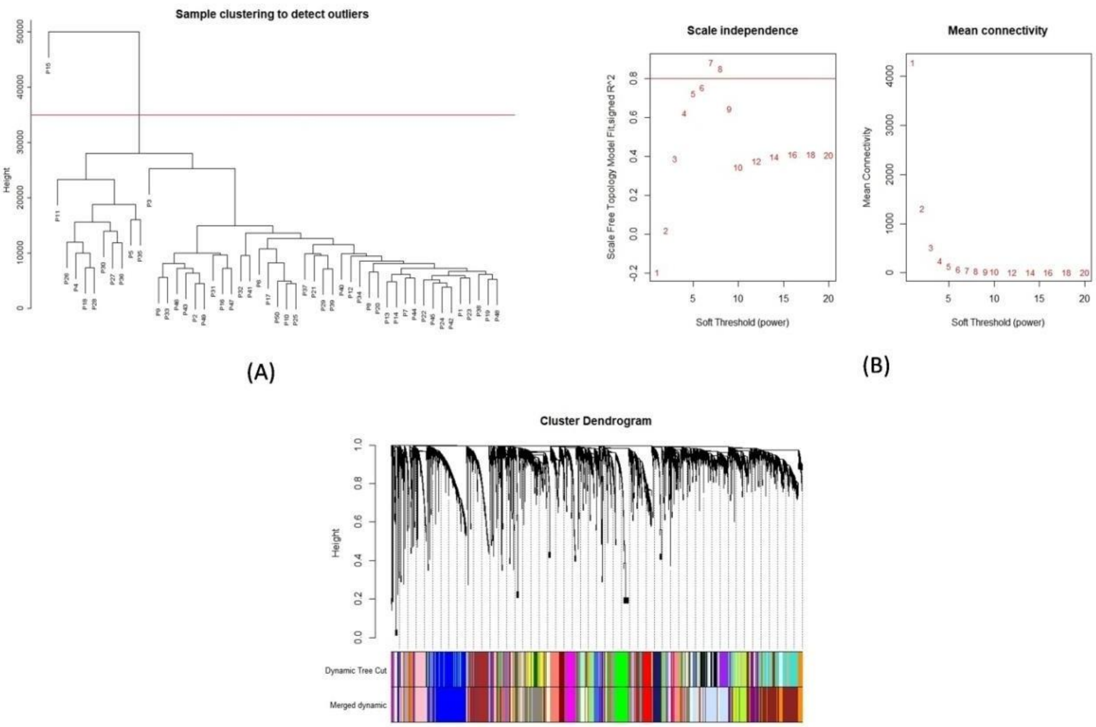

(A) (B)

(C)

Figure S4: (A – C)

Figure S4. WGCNA network and module detection in TCGA LGG Females: A) Clustering of sample data to detect outliers. B) Determining soft threshold and mean connectivity (β=7). C) Hierarchical clusteringdendrogram of the genes based on topological overlap dissimilarity. Colour coded modules of inter connected genes represents branches of clustering tree.

CGGA

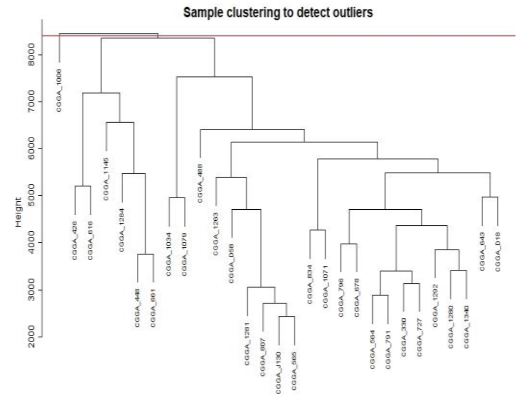

(A)

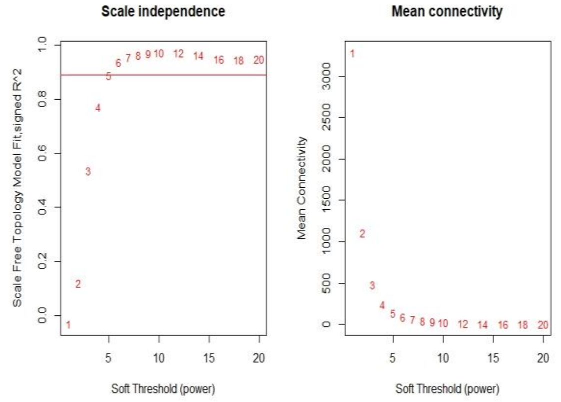

(B)

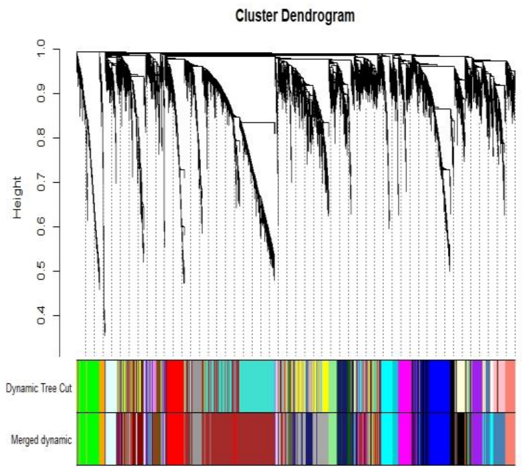

(C)

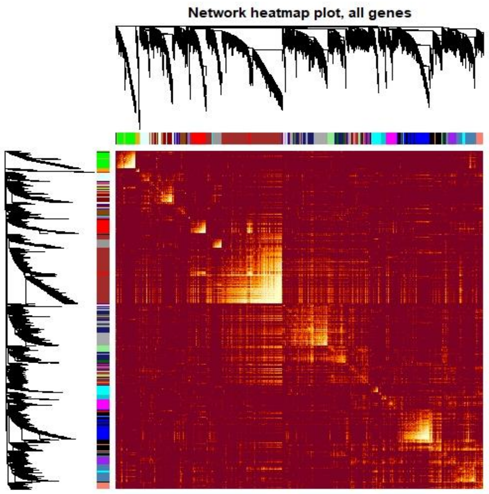

(D)

Figure S5: (A-D)

Figure S5. WGCNA network and module detection in CGGA LGG Males: A) Clustering of sample data to detect outliers. B) Determining soft threshold and mean connectivity (β=5). C) Hierarchical clustering dendrogram of the genes based on topological overlap dissimilarity. Colour coded Modules of inter connected genes represents branches of clustering tree. (D) Network heat map plot of genes in different cluster. Heat map depicts the Topological Overlap Matrix among all genes in the analysis. Dark colour represents low overlap and progressively lighter colour represents higher overlap. Blocks of lighter colour along the diagonal are the modules. (n=29 males).

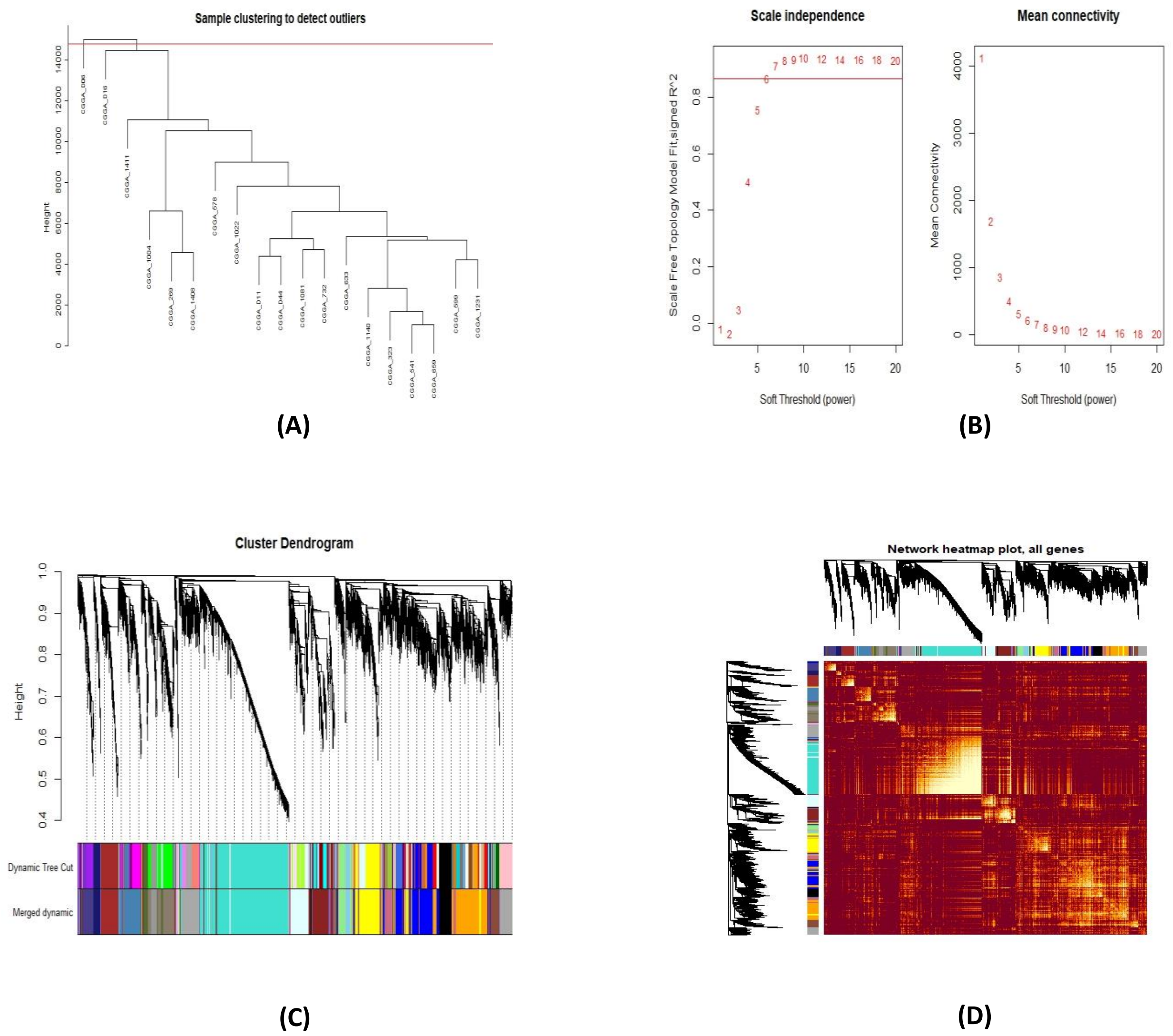

Figure S6: (A-D)

Figure S6. WGCNA network and module detection in CGGA LGG Females: A) Clustering of sample data todetect outliers. B) Determining soft threshold and mean connectivity (β=6). C) Hierarchical clusteringdendrogram of the genes based on topological overlap dissimilarity. Colour coded Modules of interconnected genes represents branches of clustering tree. (D) Network heat map plot of genes in different cluster. Heat map depicts the Topological Overlap Matrix among all genes in the analysis. Dark colour represents low overlap and progressively lighter colour represents higher overlap. Blocks of lighter colour along the diagonal are the modules. (n=19 females).

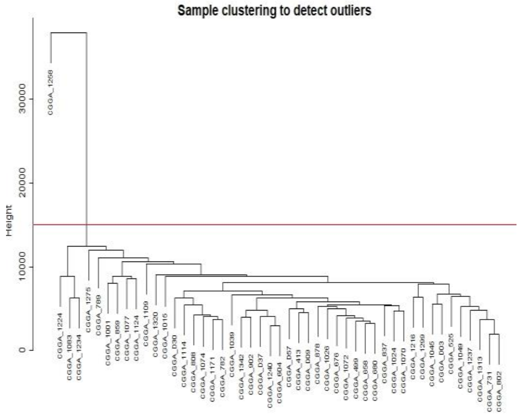

(A)

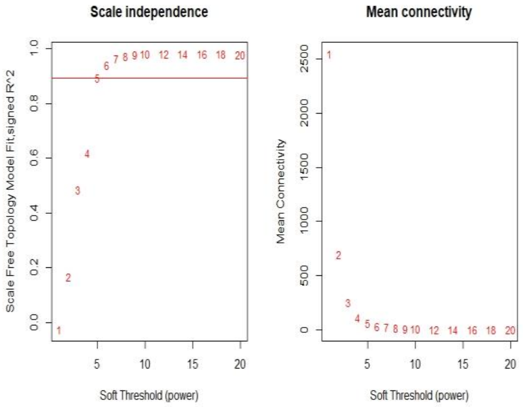

(B)

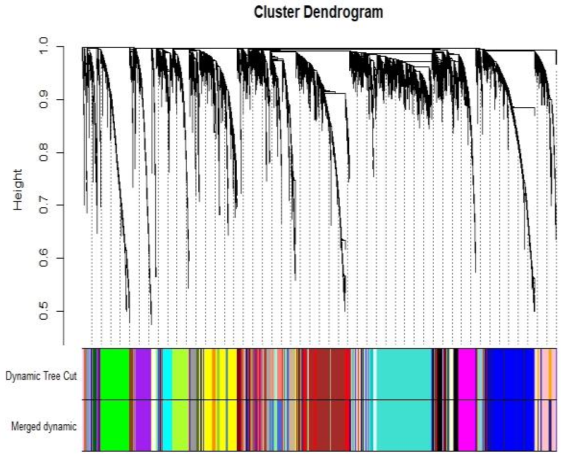

(C)

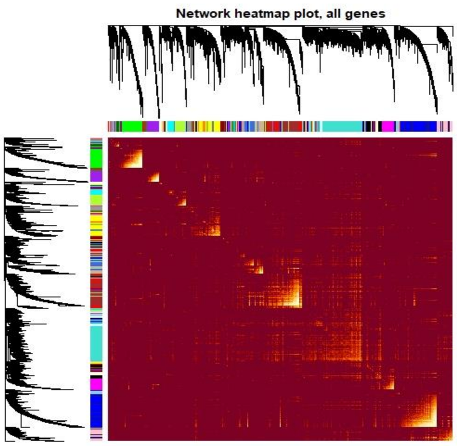

(D)

Figure S7: (A-D)

Figure S7. WGCNA network and module detection in CGGA GBM Males: A) Clustering of sample data to detect outliers. B) Determining soft threshold and mean connectivity (β=5). C) Hierarchical clustering dendrogram of the genes based on topological overlap dissimilarity. Color coded Modules of inter connected genes represents branches of clustering tree. (D) Network heat map plot of genes in different cluster. Heat map depicts the Topological Overlap Matrix among all genes in the analysis. Dark colour represents low overlap and progressively lighter colour represents higher overlap. Blocks of lighter colour along the diagonal are the modules. (n= 48 males).

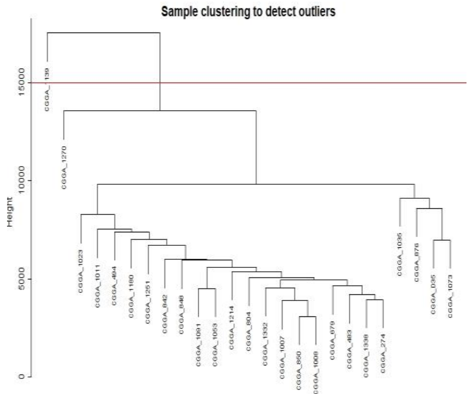

(A)

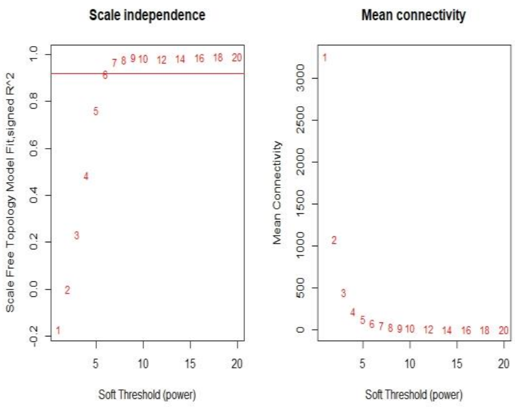

(B)

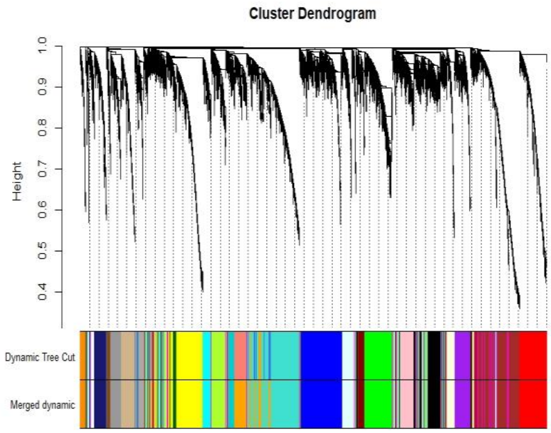

(C)

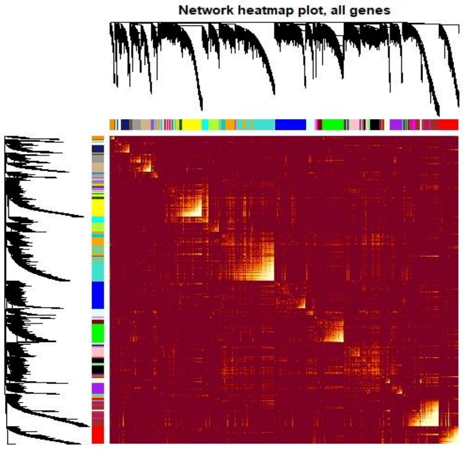

(D)

Figure S8: (A-D)

Figure S8. WGCNA network and module detection in CGGA GBM Females: A) Clustering of sample data todetect outliers. B) Determining soft threshold and mean connectivity (β=6). C) Hierarchical clusteringdendrogram of the genes based on topological overlap dissimilarity. Colour coded Modules of inter connected genes represents branches of clustering tree. (D) Network heat map plot of genes in differentcluster. Heat map depicts the Topological Overlap Matrix among all genes in the analysis. Dark colour represents low overlap and progressively lighter colour represents higher overlap. Blocks of lighter colour along the diagonal are the modules. (n= 25 females).

GBM

Module	Size	MeDC	pVal	Top_LOC
mod5	960	-0.11622	0	KIF20B, LIMS1, IREB2
mod6	721	-0.08504	0	LRRC7, MACROD2, LYPD6B
mod11	522	-0.09911	0	RHOBTB2, RHOV, REC8
mod14	321	-0.08937	0	SHANK3, SH3BGRL2, SERINC1
mod18	184	-0.13332	0	STOX2, SUPV3L1, STARD4-AS1
mod1	2038	-0.10038	0.02	B4GALT6, ANKS1B, AK3
mod2	1802	-0.08114	0.02	CDH12, CSPG4P1Y, DCAF12L2
mod3	1242	-0.09074	0.02	3912, 3890, 4053
mod8	637	-0.09808	0.02	NLGN4Y, PABPC1L2A, NXPH2
mod9	608	-0.09451	0.02	PHIP, PLCXD3, PAXBP1-AS1
mod15	320	-0.12151	0.02	SLC24A5, SLCO5A1, SLC37A4
Mod19	174	-0.13253	0.02	TACR2, SYNJ1, SYT12
Mod29	118	-0.15734	0.02	VSNL1
Mod27	141	-0.14724	0.06	UNC79, UBE4A, UNC13B
Mod30	109	-0.08365	0.06	

Figure S9

Figure S9. Table showing differentially expressed genes, their significant modules (network) obtained and their ontological significance in males over females in CGGA GBM.

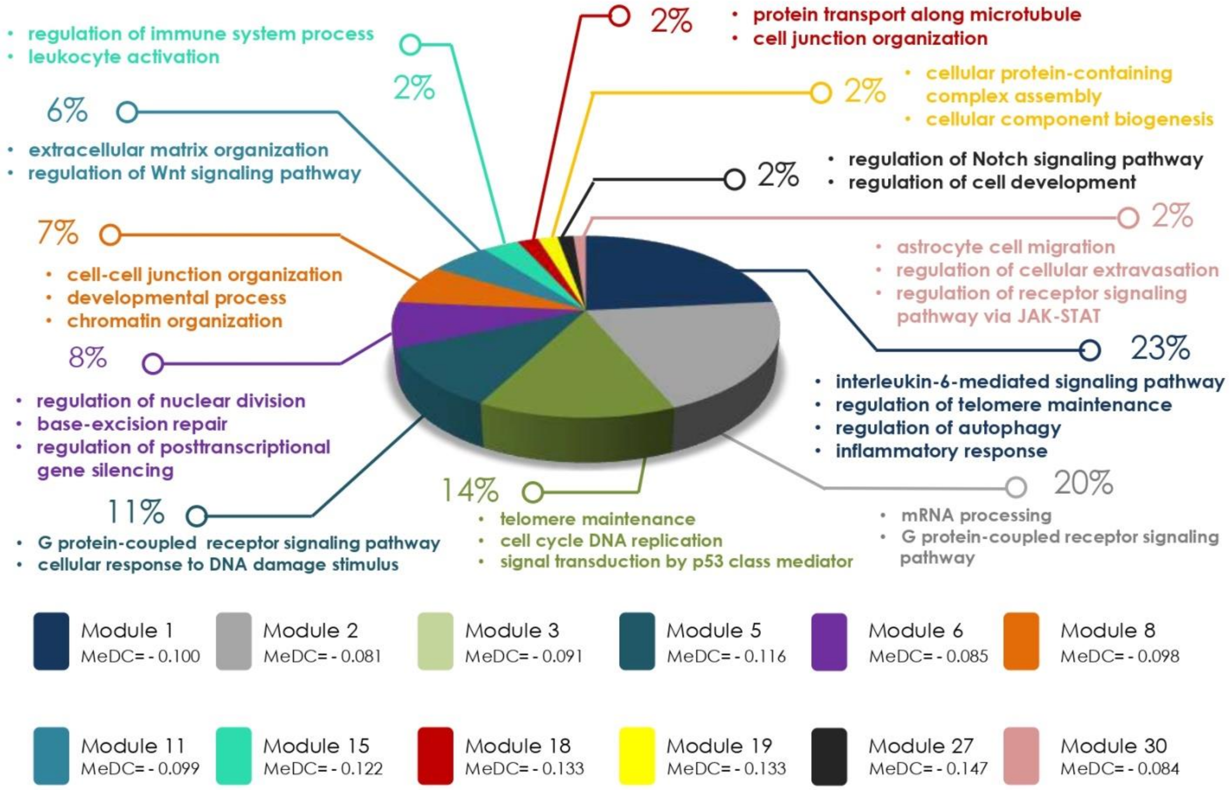

Figure S10

Figure S10. Diagram representing modules obtained in CGGA GBM after WGCNA and MDC (Modular Differential connectivity) analysis with significant MeDC (Mean Differential Connectivity) values and their ontological functions. Out of 15 modules with significant MeDC values representing LOC (Loss of connectivity) in males over females, only genes of 12 modules have significant ontological function as assessed by Gene Ontology database.
